# Pheochromocytoma presenting with severe hyperglycemia and metabolic acidosis following intra-articular glucocorticoid administration: a case report

**DOI:** 10.1186/s13256-018-1945-z

**Published:** 2019-01-05

**Authors:** Masako Tomoyasu, Yusaku Mori, Ayako Fukase, Hideki Kushima, Tsutomu Hirano

**Affiliations:** Division of Diabetes, Metabolism, and Endocrinology, Department of Internal Medicine, Showa University School of Medicine, 1-5-8 Hatanodai, Shinagawa, Tokyo, 142-8555 Japan

**Keywords:** Diabetic ketoacidosis, Fulminant type 1 diabetes, Glucocorticoid, Hyperglycemia, Pheochromocytoma

## Abstract

**Background:**

There are several reports of pheochromocytoma crisis triggered by systemic glucocorticoid administration. However, pheochromocytoma crisis after intra-articular glucocorticoid administration has been rarely reported.

**Case presentation:**

A 45-year-old Japanese man presented to our hospital with a sudden, severe headache. He had no history of diabetes. He had received an intra-articular injection of betamethasone (2 mg) for joint pain, 2 days prior to his admission. On examination, his blood pressure was 240/126 mmHg and pulse was 120 beats/minute. The possibility of cerebrovascular events was ruled out by imaging studies and lumbar puncture. Blood tests revealed severe hyperglycemia (523 mg/dL) and metabolic acidosis (pH 7.21, anion gap 26.2 mEq/L, lactate 11.75 mmol/L) with a glycosylated hemoglobin level of 5.7%. Although a urine sample could not be obtained, fulminant type 1 diabetes mellitus and diabetic ketoacidosis were suspected based on these findings. However, after the initial treatment for diabetic ketoacidosis, his insulin secretion was found to be normal and the plasma levels of ketones were not elevated. This excluded the possibility of fulminant type 1 diabetes mellitus and diabetic ketoacidosis. Subsequently, a left adrenal gland tumor and elevated levels of serum catecholamine and urinary catecholamine metabolites were detected, while his other hormone levels were normal. Serum catecholamine levels did not decrease following the clonidine test, and a functional scintigraphy using iodine-131 metaiodobenzylguanidine showed strong uptake in the region of the left adrenal gland. Although no signs of pheochromocytoma crisis, such as paroxysmal hyperglycemia and hypertension, had been observed since admission, a pheochromocytoma was diagnosed based on the investigations. After controlling his blood pressure, a left adrenalectomy was performed.

**Conclusions:**

This case illustrates that intra-articular glucocorticoid administration can induce a pheochromocytoma crisis and an increase in hyperglycemia, and that pheochromocytoma crisis can resemble the clinical picture of fulminant type 1 diabetes mellitus owing to severe hyperglycemia with metabolic acidosis and normal glycosylated hemoglobin levels, especially under the influence of glucocorticoid.

## Background

Pheochromocytoma is a catecholamine-secreting tumor that arises from the chromaffin cells of the adrenal medulla or from the paragangliomas of the extra-adrenal gland. One of the classical symptoms of pheochromocytoma crisis is hyperglycemia [1] that might be caused by increased insulin resistance in peripheral tissues and impaired insulin secretion [2]. However, the frequency of severe hyperglycemia (for example, plasma glucose levels > 400 mg/dL) induced by pheochromocytoma crisis is largely unknown.

There are many reports of pheochromocytoma crisis triggered by systemic glucocorticoid administration [3]. Intra-articular glucocorticoid administration is a common practice for joint pains, and is considered much safer than systemic administration, which can lead to unfavorable metabolic changes including hyperglycemia.

We report a case of pheochromocytoma crisis following an intra-articular glucocorticoid injection. It was initially treated as diabetic ketoacidosis (DKA) induced by fulminant type 1 diabetes mellitus (FT1DM) due to the presence of severe hyperglycemia and metabolic acidosis with normal glycosylated hemoglobin (HbA1c) level.

## Case presentation

A 45-year-old Japanese man received an intra-articular injection of glucocorticoid (betamethasone 2 mg) for pain in his right elbow joint 2 days prior to admission. On the day of admission, he experienced general fatigue. Two hours later, he experienced a sudden, severe headache and was brought to our emergency department in an ambulance.

He was diagnosed as having hypertension at 44 years of age, and his blood pressure was under control with lisinopril 10 mg/day. He had no other significant past medical history or any episodic headaches. He was a tobacco smoker (20 cigarettes/day) for the past 24 years, and consumed approximately 50–100 g/day of alcohol, but was not addicted to any drugs, such as cocaine. He was married and had two children (a daughter, 12-years old; a son, 1-year old). His family had no history of diabetes, cancer, or any endocrine diseases, like pheochromocytoma, medullary thyroid carcinoma, parathyroid adenoma or hyperplasia, mucosal neuroma, and kidney cancer.

His vital signs were as follows: blood pressure, 240/126 mmHg; pulse, 120 beats/minute (regular); temperature, 37.6 °C; respiratory rate, 25 breaths/minute. Except for excessive perspiration and sinus tachycardia, physical and neurological examinations showed no significant findings, such as pallor, tremor, enlarged thyroid gland or palpable thyroid nodule, enlarged lymph nodes, abnormal lung or heart sounds, meningeal irritation, and central or peripheral nerve dysfunction. Initially, subarachnoid hemorrhage was suspected due to severe headache and elevated blood pressure. However, computed tomography and magnetic resonance images of his head were normal. In addition, the cerebrospinal fluid drawn by lumbar puncture was clear, eliminating the possibility of cerebral vascular diseases, including subarachnoid hemorrhage. The results of the initial laboratory tests are shown in Table 1. Based on severe hyperglycemia and metabolic acidosis with normal HbA1c level on investigations, we suspected DKA caused by FT1DM.

**Table 1:** Laboratory data

Blood tests at admission	
Complete blood count	
White blood cell	19,600/μL
Red blood cell	509 × 10^4^/μL
Hemoglobin	16.1 g/dL
Hematocrit	47.30%
Platelet	12.9 × 10^4^/μL
Arterial blood gas (oxygen 6 l/minute)	
pH	7.21
PaCO_2_	30.2 mmHg
PaO_2_	138.9 mmHg
Hydrogen carbonate	11.8 mmoL/L
Base excess	−14.6 mmoL/L
Lactate	11.75 mmoL/L
Anion gap	26.2 mEq/L
Additional tests for differential diagnosis	
Blood test	
Glucose	109 mg/dL
Insulin	7.8 μU/mL
C peptide	2.64 ng/mL
GAD antibody	< 0.5 U/mL
ACTH	23.1 pg/mL
Cortisol	14.6 μg/dL
DHEA-S	372 ng/mL
Plasma renin activity	6.1 ng/mL/hour
Aldosterone	162.3 pg/mL
Growth hormone	0.10 ng/mL
IGF-1	211 ng/mL
TSH	1.59 μIU/mL
Free triiodothyronine	2.63 pg/mL
Free thyroxin	0.93 ng/dL
Biochemistry	
Total bilirubin	0.9 mg/dL
Aspartate transaminase	47 IU/L
Alanine aminotransferase	59 IU/L
Creatine kinase	215 IU/L
Blood urea nitrogen	16.9 mg/dL
Creatinine	1.35 mg/dL
Sodium	138.6 mEq/L
Potassium	3.1 mEq/L
Chloride	96.9 mEq/L
Glucose	523 mg/dL
Hemoglobin A1c	5.70%
Total ketone body	289 μmoL/L
Acetoacetic acid	54 μmoL/L
3-hydroxybutyric acid	235 μmoL/L
Urinary test	
Metanephrine	1.87 mg/day
Normetanephrine	0.83 mg/day
Adrenalin	322.4 μg/day
Noradrenaline	347.6 μg/day
Dopamine	1003.9 μg/day
Clonidine (0.15 mg) suppression test	
Baseline	
Adrenaline	0.15 ng/mL
Noradrenaline	0.31 ng/mL
Dopamine	≦ 0.01 ng/mL
3 hours after clonidine	
Adrenaline	0.11 ng/mL
Noradrenaline	0.27 ng/mL
Dopamine	≦ 0.01 ng/mL

*ACTH* adrenocorticotropic hormone, *DHEA-S* dehydroepiandrosterone sulfate, *GAD* glutamic acid decarboxylase, *IGF-1* insulin-like growth factors-1, *PaCO*_*2*_ partial pressure of arterial carbon dioxide, *PaO*_*2*_ partial pressure of arterial oxygen, *TSH* thyroid-stimulating hormone

**Fig. 1:**
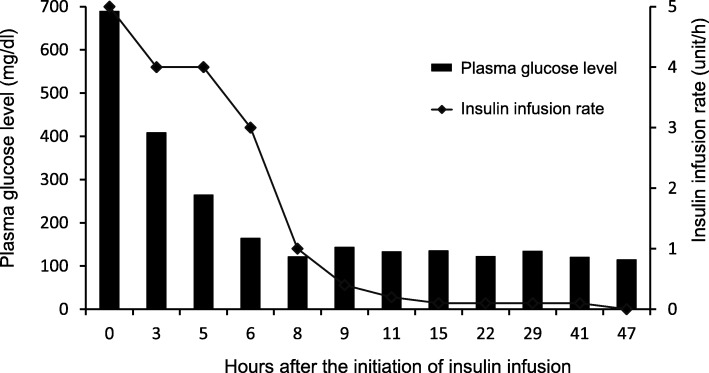
Course of plasma glucose levels and insulin infusion rate. *Bars* show plasma glucose levels and the *line* indicates insulin injection rate at indicated hours after the treatment

**Fig. 2:**
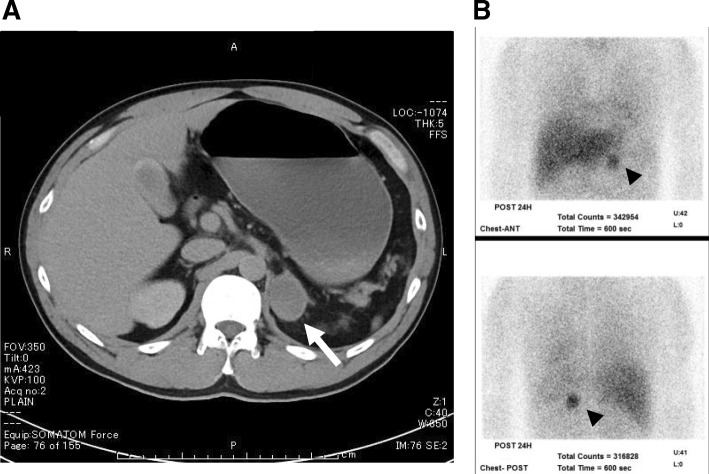
Images of abdominal computed tomography and functional scintigraphy using iodine-131 metaiodobenzylguanidine. **a** Abdominal computed tomography, the *arrow* indicates a left adrenal gland tumor. **b** Anterior (*upper*) and posterior (*lower*) reprojected images of iodine-131 metaiodobenzylguanidine scintigraphy, the *arrow head* indicates strong uptake in the region of the left adrenal gland

**Fig. 3:**
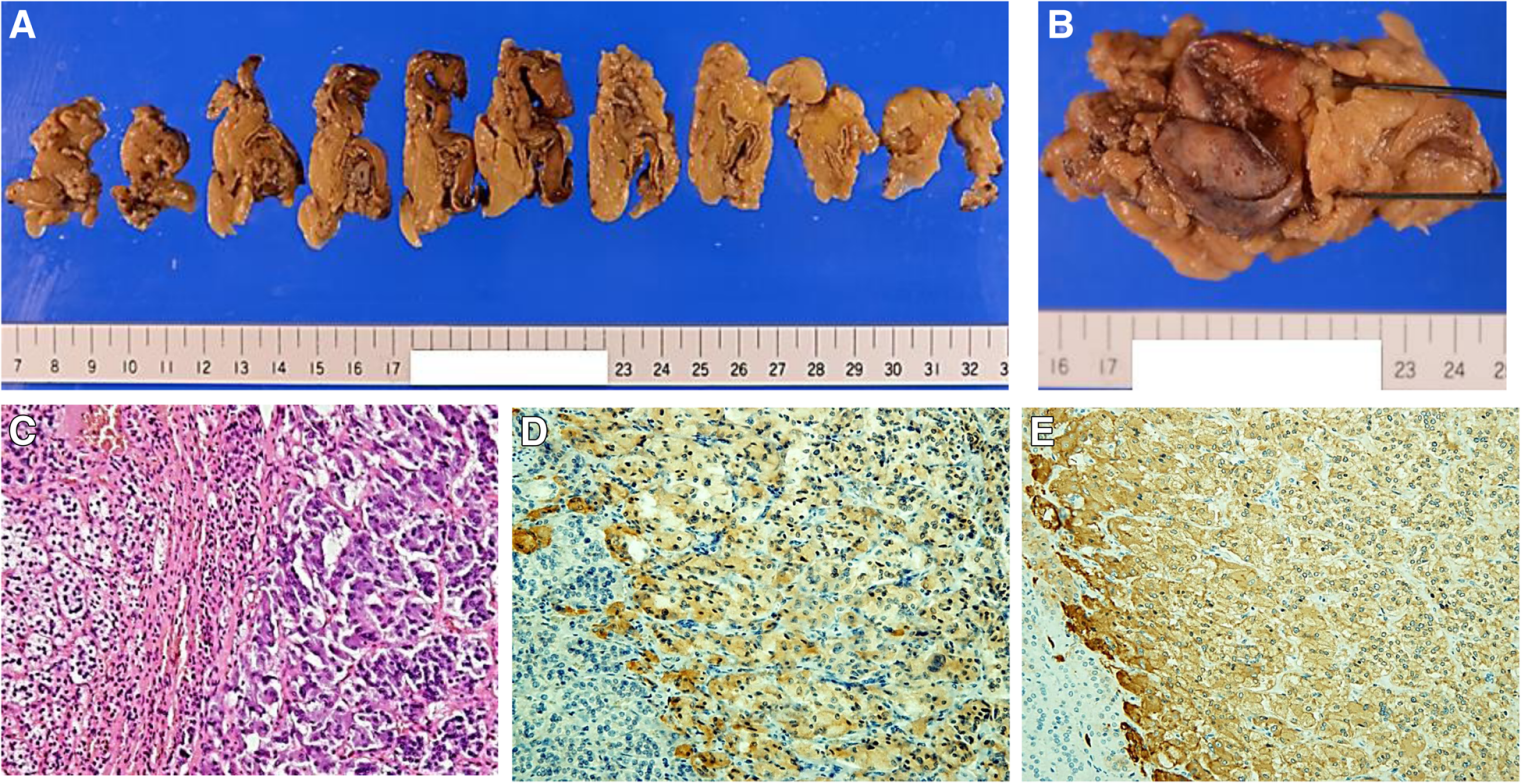
A left adrenal gland tumor removed by adrenalectomy. **a**, **b** Gross pathology of the tumor of the left adrenal gland. **c**–**e** Microscopic image of the tumor under hhematoxylin and eosin staining (**c**), chromogranin A immunostaining (**d**), or synaptophysin immunostaining (**e**). The magnification was 200×. **d**, **e** The cells stained in brown are positive for chromogranin A and synaptophysin, respectively

We initiated the standard treatment for DKA, including intravenous insulin infusion and fluid replacement. The course of insulin infusion rates and plasma glucose levels is presented in Fig. 1. Following the initiation of insulin infusion, his plasma glucose level rapidly decreased and recovered to normal within 2 hours. In 18 hours, the lowest insulin infusion rate (0.1 U/h) was required to maintain normoglycemia. At the same time, our investigations showed that his basal insulin secretion was normal, and plasma ketone levels were not elevated, as shown in Table 1. These findings indicated metabolic acidosis induced by lactic acid, and excluded the possibility of FT1DM. Subsequently, he was screened for secondary diabetes. A left adrenal gland tumor (3 cm in diameter) was detected by abdominal computed tomography (Fig. 2a). The levels of urinary catecholamine metabolites (metanephrine and normetanephrine) and serum catecholamines were significantly elevated; however, the other hormone levels were normal (Table 1). Elevated levels of serum adrenaline and noradrenaline did not reduce following the clonidine test, which indicated an autonomic catecholamine secretion. A functional scintigraphy of the adrenal gland using iodine-131 metaiodobenzylguanidine showed a strong uptake in the region of the left adrenal gland (Fig. 2b). These findings led to a diagnosis of pheochromocytoma. However, there were no findings suggestive of medullary thyroid carcinoma, parathyroid adenoma or hyperplasia, or any other endocrine diseases. His blood pressure was controlled with an α1 adrenergic receptor blocker doxazosin (12 mg/day), following which a left adrenalectomy was performed 85 days after his admission. The tumor size was approximately 118.75 cm^3^ (9.5 cm × 5 cm × 2.5 cm, Fig. 3a, b). On histopathological assessment, most tumor cells were positive for chromogranin A and synaptophysin, which were consistent with the diagnosis of pheochromocytoma (Fig. 3c–e) [4]. There were no signs of lymphovascular or capsular invasion. However, markers of malignancy, such as Ki-67 labeling index and Pheochromocytoma of the Adrenal Gland Scaled Score indicated borderline abnormalities (1.5% and 4, respectively), which required careful follow-up [5, 6]. Since this first event, he showed no signs of pheochromocytoma crisis such as paroxysmal hyperglycemia and hypertension. During the postoperative follow-up for 28 months, he did not show any symptoms or signs indicating recurrence of pheochromocytoma.

## Discussion

We encountered a case of pheochromocytoma crisis, which was possibly triggered by an intra-articular administration of glucocorticoid, 2 days prior to the crisis. Furthermore, it might have contributed to the development of severe hyperglycemia during the pheochromocytoma crisis. Accompanied by metabolic acidosis and normal HbA1c levels, the severe hyperglycemia misled us to a diagnosis of DKA induced by FT1DM.

One case of pheochromocytoma crisis induced by intra-articular glucocorticoid injection has been reported in the literature [7], in which the pheochromocytoma crisis developed 1 day after an injection of dexamethasone into the shoulder joint (the dose is not mentioned). In our case, the pheochromocytoma crisis developed 2 days after a dexamethasone injection into the elbow joint. A previous study demonstrated the kinetics of glucocorticoid delivered through an intra-articular injection [8]. In patients who received a dose of methylprednisolone acetate 40 mg, serum levels of methylprednisolone were still elevated 3 days after the intra-articular injections. Thus, it is reasonable to assume that the intra-articular glucocorticoid injection triggered the pheochromocytoma crisis in this case.

In the pheochromocytoma crisis in our case, severe hyperglycemia with metabolic acidosis and normal HbA1c levels were observed, due to which we initially suspected DKA induced by FT1DM. FT1DM, a subtype of T1DM, is characterized by an extremely rapid process of β-cell destruction [9]. In most cases of FT1DM, HbA1c levels are normal during DKA. In our patient, who had no history of diabetes, the intra-articular glucocorticoid injection probably enhanced the hyperglycemia during the pheochromocytoma crisis. Furthermore, metabolic acidosis induced by lactic acid has been reported in pheochromocytoma crisis [10]. When these changes occur simultaneously, the pheochromocytoma crisis can show findings similar to FT1DM-induced DKA. However, DKA induced by pheochromocytoma crisis has been rarely reported [11, 12]. It is possible that this case would have developed DKA if insulin therapy had not been administered. DKA is a life-threatening condition; hence, the initial treatment should not be delayed when suspected, especially when the differential diagnosis is uncertain.

## Conclusion

There are two important lessons from this case. First, an intra-articular injection of glucocorticoid can be a possible cause of a pheochromocytoma crisis. Second, pheochromocytoma crisis occurring under the influence of glucocorticoid can have clinical findings similar to DKA induced by FT1DM.

## Abbreviations

DKA: Diabetic ketoacidosis
FT1DM: Fulminant type 1 diabetes mellitus
HbA1c: Glycosylated hemoglobin
